# Isolation and functional analysis of fatty acid desaturase genes from peanut (*Arachis hypogaea* L.)

**DOI:** 10.1371/journal.pone.0189759

**Published:** 2017-12-15

**Authors:** Xiaoyuan Chi, Zhimeng Zhang, Na Chen, Xiaowen Zhang, Mian Wang, Mingna Chen, Tong Wang, Lijuan Pan, Jing Chen, Zhen Yang, Xiangyu Guan, Shanlin Yu

**Affiliations:** 1 Shandong Peanut Research Institute, Qingdao, Shandong, P. R. China; 2 Yellow Sea Fisheries Research Institute, Chinese Academy of Fishery Sciences, Qingdao, Shandong, P. R. China; 3 School of Ocean Sciences, China University of Geosciences, Beijing, P. R. China; Texas Tech University, UNITED STATES

## Abstract

**Background:**

Fatty acid desaturases are enzymes that introduce double bonds into fatty acyl chains. Extensive studies of fatty acid desaturases have been done in many plants. However, less is known about the diversity of this gene family in peanut (*Arachis hypogaea* L.), an important oilseed crop that is cultivated worldwide.

**Results:**

In this study, twelve novel *AhFADs* genes were identified and isolated from peanut. Quantitative real-time PCR analysis indicated that the transcript abundances of *AhFAB2-2* and *AhFAD3-1* were higher in seeds than in other tissues examined, whereas the *AhADS* and *AhFAD7-1* transcripts were more abundant in leaves. *AhFAB2-3*, *AhFAD3-2*, *AhFAD4*, *AhSLD-4*, and *AhDES* genes were highly expressed in flowers, whereas *AhFAD7-2*, *AhSLD-2*, and *AhSLD-3* were expressed most strongly in stems. During seed development, the expressions of *AhFAB2-2*, *AhFAD3-1*, *AhFAD7-1*, and *AhSLD-3* gradually increased in abundance, reached a maximum expression level, and then decreased. The *AhFAB2-3*, *AhFAD3-2*, *AhFAD4*, *AhADS*, and *AhDES* transcript levels remained relatively high at the initial stage of seed development, but decreased thereafter. The *AhSLD-4* transcript level remained relatively low at the initial stage of seed development, but showed a dramatic increase in abundance at the final stage. The *AhFAD7-2* and *AhSLD-2* transcript levels remained relatively high at the initial stage of seed development, but then decreased, and finally increased again. The *AhFAD* transcripts were differentially expressed following exposure to abiotic stresses or abscisic acid. Moreover, the functions of one *AhFAD6* and four *AhSLD* genes were confirmed by heterologous expression in *Synechococcus elongates* or *Saccharomyces cerevisiae*.

**Conclusions:**

The present study provides valuable information that improves understanding of the biological roles of *FAD* genes in fatty acid synthesis, and will help peanut breeders improve the quality of peanut oil via molecular design breeding.

## Introduction

Peanut (*Arachis hypogaea* L.) is an allotetraploid species (2n = 4x = 40, AABB) cultivated widely in tropical, subtropical and temperate regions [1]. The peanut seed is comprised of around 50% oil, of which approximately 80% is oleic (36–67%) and linoleic (15–43%) acids [2]. A high O/L ratio (ratio of oleic and linoleic acid) is the most desired oil quality trait as it increases shelf life and the health benefits to manufacturers and consumers, respectively [3]. Improvements in peanut oil content and quality traits (high oleic and low linoleic acid) could be accelerated by investigating the fatty acid biosynthesis pathway.

Fatty acid desaturases are responsible for the insertion of double bonds into pre-formed fatty acid chains, and play an essential role in fatty acid metabolism and the maintenance of biological membranes in living organisms [4]. They belong to a large gene family that contains conserved histidine regions. Histidine-rich boxes are thought to form a part of the diiron center where oxygen activation and substrate oxidation occur [5, 6].

For higher plants, most information on the function and specificity of fatty acid desaturases has come from characterization of *Arabidopsis* mutants that were deficient in specific desaturation activities [7]. The desaturase genes detected in *Arabidopsis* are divided into several subfamilies: stearoyl-ACP desaturase (FAB2), microsomal Δ12 desaturase (FAD2), plastidial Δ12 desaturase (FAD6), microsomal ω3 desaturase (FAD3), plastidial ω3 desaturase (FAD7), plastidial ω3 desaturase (FAD8), trans Δ3 desaturase (FAD4), Δ7 desaturase (FAD5), Δ9 desaturase (ADS), sphingolipid Δ8 desaturase (SLD), and sphingolipid Δ4 desaturase (DES). FAB2 is the only soluble desaturase that has been characterized to date, and it catalyzes the desaturation of stearic acid (C18:0) to C18:1 in the acyl carrier protein (ACP)-bound form [8]. FAD2 and FAD6 are ω6 desaturases that synthesize dienoic linoleic fatty acid (C18:2) from oleic acid (C18:1) in the endoplasmic reticulum (ER) and plastids, respectively. FAD3, FAD7, and FAD8 are ω3 desaturases that synthesize linolenic (C18:3) from linoleic (C18:2) acid in the ER (FAD3) and plastids (FAD7 and FAD8) [9, 10]. FAD4 and FAD5 specifically produce C16:1 from C16:0 for PG and MGDG, respectively [8]. ADS is a Δ9 acyl-lipid desaturase that participates in desaturation at the Δ9 position of C16:0 in the ER [11, 12]. SLD encodes a sphingolipid Δ8 desaturase that leads to the accumulation of 8 (Z/E)-C18-phytosphingenine in the leaves and roots of *Arabidopsis* plants [13, 14]. DES encodes the sphingolipid Δ4 desaturase responsible for the synthesis of Δ4-unsaturated LCBs, such as sphingosine and sphinga-4,8-dienine in *Arabidopsis* [15, 16].

To date, several types of fatty acid desaturase genes have been cloned and characterized from peanut, including *AhFAB2-1*, *AhSLD-1*, four microsomal *AhFAD2* and two chloroplast *AhFAD6* genes [17, 18]. Among them, *FAD2* is the most well-studied fatty acid desaturase gene. Two microsomal oleoyl-PC desaturase genes (*AhFAD2-1A* and *AhFAD2-1B*), each having its origin in different diploid progenitor species, have been isolated from cultivated peanut [19–21]. Reduction in transcript levels or inactivation of both genes is required to produce high O/L genotypes [22–26]. Different types of DNA markers from these two genes have been developed to facilitate marker-assisted selection for the high-oleate trait [27–30]. The functions of *AhFAD2-2* and *AhFAD6* were verified by heterologous expression in *S*. *cerevisiae*. Linoleic acid (18:2), normally not present in wild-type yeast cells, was detected in transformants of these two genes [17].

The cultivated peanut (*Arachis hypogaea* L.) is derived from two wild diploid species *Arachis duranensis* (A genome) and *Arachis ipaensis* (B genome) [31, 32]. Now the genome sequences of *Arachis duranensis* and *Arachis ipaensis* were released. The availability of these genomes will lead to further advances in knowledge of genetic changes since the very recent polyploidization event that gave rise to cultivated peanut and to an expanding knowledge of understudied areas of plant biology [33, 34]. In this study, we isolated twelve novel desaturase genes from cultivated peanut. The expression patterns of these genes were investigated in different tissues and at different stages of seed development. The expression of *FAD* genes in response to abiotic stress and abscisic acid (ABA) was also analyzed. Additionally, the functions of *AhFAD6* and *AhSLDs* were confirmed by heterologous expression in *Synechococcus elongatus* (strain PCC 7942) or yeast (*Saccharomyces cerevisiae*). Our results indicated that these two types of genes are strong candidates for modifying fatty acid biosynthesis in peanut.

## Results and discussion

### Isolation of *FAD* genes from peanut

In a previous study, four fatty acid desaturases were isolated from peanut. These were *AhFAB2-1*, *AhFAD2-2*, *AhFAD6*, and *AhSLD-1* [17]. In this study, twelve new genes that probably encode *FAD* proteins were found using Bioedit software [35]. They were cloned and designated as *AhFAB2-2*, *AhFAB2-3*, *AhFAD3-1*, *AhFAD3-2*, *AhFAD4*, *AhADS*, *AhFAD7-1*, *AhFAD7-2*, *AhDES*, *AhSLD-2*, *AhSLD-3*, and *AhSLD-4*, according to the homologous genes identified in *Arabidopsis* (Table 1). Among the twelve genes, six had complete open reading frames in the peanut cDNA library and were cloned by conventional RT-PCR. However, six genes were cloned using the rapid amplification of cDNA ends (RACE) method. The open reading frames of these genes ranged from 924 bp to1371 bp in length, and encoded 307 to 456 amino acids (Table 1 and S1 Table). The sequence information for the twelve genes was submitted to Genbank along with their identification numbers (Table 1).

**Table 1:** Fatty acid desaturase genes in peanut.

Protein	Accession	Len (aa)	ORF(bp)	5’ upstream region (bp)	3’ downstream region (bp)	Molecular mass (kDa)	PI
FAB2-1[17]	FJ230310	406	1221	19	259	46.2516	6.24
FAB2-2	KF358459	385	1158	143	226	43.6779	5.69
FAB2-3	KF358460	395	1188	56	208	44.8906	6.37
FAD2-1A[19,20]	AAB84262	379	1140	79	137	43.6513	9.03
FAD2-1B[19,20]	AAF82293	379	1140	0	0	43.6363	8.87
FAD2-2[17]	FJ768732	383	1152	97	247	43.8292	8.8
FAD6[17]	FJ768730	442	1329	96	264	51.642	9.09
FAD3-1	KF516546	376	1131	160	263	43.8524	8.95
FAD3-2A	KF516547	392	1179	155	125	46.2278	7.52
FAD3-2B	KF516548	388	1167	155	125	45.6072	7.51
FAD3-2C	KF516549	388	1167	155	125	45.6372	7.51
FAD4A	KF516555	307	924	1	228	34.5927	8.76
FAD4B	KF516545	307	924	1	228	34.5666	8.76
ADS	KF516550	393	1182	23	76	45.2418	9.29
FAD7-1A	KF516551	456	1371	455	322	52.1818	7.17
FAD7-1B	KF516552	453	1362	455	322	51.7944	8.08
FAD7-2	KF516553	455	1368	279	253	51.6349	8.23
DES	KF516554	334	1005	138	211	38.8998	7.87
SLD-1[17]	FJ824607	448	1347	17	433	51.3642	9.06
SLD-2	KF358457	448	1347	287	185	51.9188	8.41
SLD-3	KF358458	448	1347	80	101	51.7837	8.35
SLD-4	KF358461	452	1359	108	428	51.8077	8.68

A search using NCBI BLAST revealed that the FAD proteins have high sequence similarities with FADs in Arabidopsis. AhFAB2-2 and AhFAB2-3 shared 64.7% identity, which was higher than that with AhFAB2-1 and AtFAB2-1. AhFAD3-1 shared about 74% sequence identities with AhFAD3-2A, AhFAD3-2B, and AhFAD3-2C. They also shared high sequence identities of about 66% with Arabidopsis AtFAD3. AhFAD4A shared a high sequence identity of 99.6% with AhFAD4B, and both of them shared 53%, 50%, and 48% similarity with AtFAD4-1, AtFAD4-2, and AtFAD4-3, respectively. AhADS shared 62.7%, 44%, and 41% amino acid sequence identities with AtFAD5, AtADS-1, AtADS-2, respectively. The AhFAD7-2 protein was most similar to AhFAD7-1A (76.8%) and AhFAD7-1B (76.8%). AhFAD7-1A, AhFAD7-1B, and AhFAD7-2 showed more than 66% identity with AtFAD7 and AtFAD8. AhDES showed 76% sequence similarity with AtDES. AhSLD-2 shares 61.2%, 94.8%, and 61.3% amino acid sequence identities with AhSLD-1, AhSLD-3, and AhSLD-4, respectively. AhSLD-1, AhSLD-2, AhSLD-3, and AhSLD-4 showed 60.3%, 72.2%, 72.2%, and 61.7% identity, respectively, with AtSLD-1, and 59.8%, 73.1%, 72.4%, and 61.9% identity to AtSLD-2, respectively.

Prediction of subcellular location by two programs, TargetP Server and Predotar, suggested that the AhFAD7-1A, AhFAD7-1B, and FAD7-2 proteins were probably located in the chloroplast, which is the same as AhFAB2-1 and AhFAD6. The first 73 amino acids at the N-terminal end of the deduced proteins for these three genes had a high proportion of hydroxylated and small, hydrophobic amino acids, which is typical of chloroplast transit peptides.

The twelve desaturase genes contained typical histidine-rich boxes (S1–S3 Figs), which was in accordance with the standard for different types of desaturase genes. For example, the two histidine-boxes of *AhFAB2-1*, *AhFAB2-2*, and *AhFAB2-3* genes were consistent with those of plastidial stearoyl-ACP desaturases, which are represented as EENRHG and DEKRHE (S1 Fig). The three histidine-boxes of four microsomal Δ15 fatty acid desaturases (*AhFAD3-1*, *AhFAD3-2A*, *AhFAD3-2B*, and *AhFAD3-2C*) and three plastidial Δ15 fatty acid desaturases (*AhFAD7-1A*, *AhFAD7-1B*, and *AhFAD7-2*) genes matched the standards for Δ15 desaturase, i.e. LGHDCGH, HR(K)THH, and HVIHH. The third histidine box of *AhSLD-1*, *AhSLD-2*, *AhSLD-3*, and *AhSLD-4* contained a His to Gln substitution at the third histidine residue, which is also found in several fatty acid desaturases, such as the plant and animal fatty acid Δ5- and Δ6-desaturases [13, 36]. Furthermore, in common with other desaturases of this type, four *AhSLD* genes encoded proteins with a cytochrome b_5_-like hem-binding domain at the N-terminus. The presence of this binding domain was characterized by the His-Pro-Gly-Gly motif, which indicated that this putative desaturase existed as a fusion protein. Sphingolipid Δ4 desaturase activity has evolved independently of sphingolipid Δ8-desaturase activity [16]. The sphingolipid Δ4 desaturases shared only limited similarity with other proteins characterized by the histidine box motifs (LAIHELSH, HLEHH, HNEHH), and they didn’t contain a cytochrome b5 domain (S2 Fig).

FAD4 encodes a predicted integral membrane protein that appears to be unrelated to classic membrane bound fatty acid desaturases, based on overall sequence conservation. Its inferred primary sequence has little resemblance to that of known fatty acid desaturases beyond the presence of histidine motifs and membrane-spanning domains. However, the FAD4 protein contains two histidine motifs resembling those of fatty acid desaturases [37]. The *AhFAD4A* and *AhFAD4B* protein sequences contained two histidine motifs, HAWAH and HSAHH (S3 Fig), whose sequence and spacing are reminiscent of, but not identical to, conserved motifs in membrane-bound desaturases [38]. While *AhFAD4A* and *AhFAD4B* contained a third histidine motif (QGHH; S3 Fig), its sequence diverged from the third histidine motif present in membrane-bound desaturases. Although glutamine substituted for histidine in these motifs [36], known membrane-bound desaturases typically have two-to-three amino acids between the glutamine and the histidines [39]. Another difference is that in characterized membrane-bound desaturases, two histidine motifs are located between the second and the third membrane spanning domains, while the third is located at the C-terminus of the protein. In *AhFAD4A* and *AhFAD4B*, only one proposed histidine motif is located between membrane spanning domains II and III, while the other two are located at the C-terminus (S3 Fig). Based on these sequence features, it seems likely that FAD4 is a metalloenzyme that evolved independently from the characterized desaturases [37].

### Phylogenetic analysis

**Fig 1:**
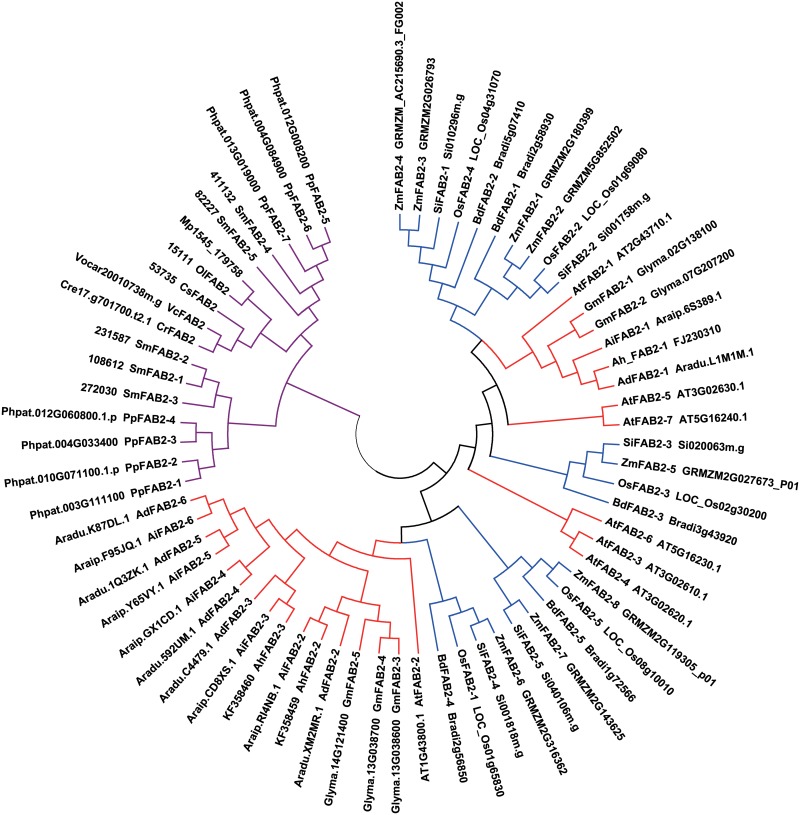
Phylogenetic tree of stearoyl-ACP desaturase gene families reconstructed by the neighbor-joining (NJ) method. Gene sequences were shown by their strain names, accession numbers (locus tags), or labels. Colored branches indicated different groups of proteins. Red: eudicot, blue: monocotyledon, purple: fern, moss and algae. Bootstrapping with 1,000 replicates was used to establish the confidence limits of the tree branches.

**Fig 2:**
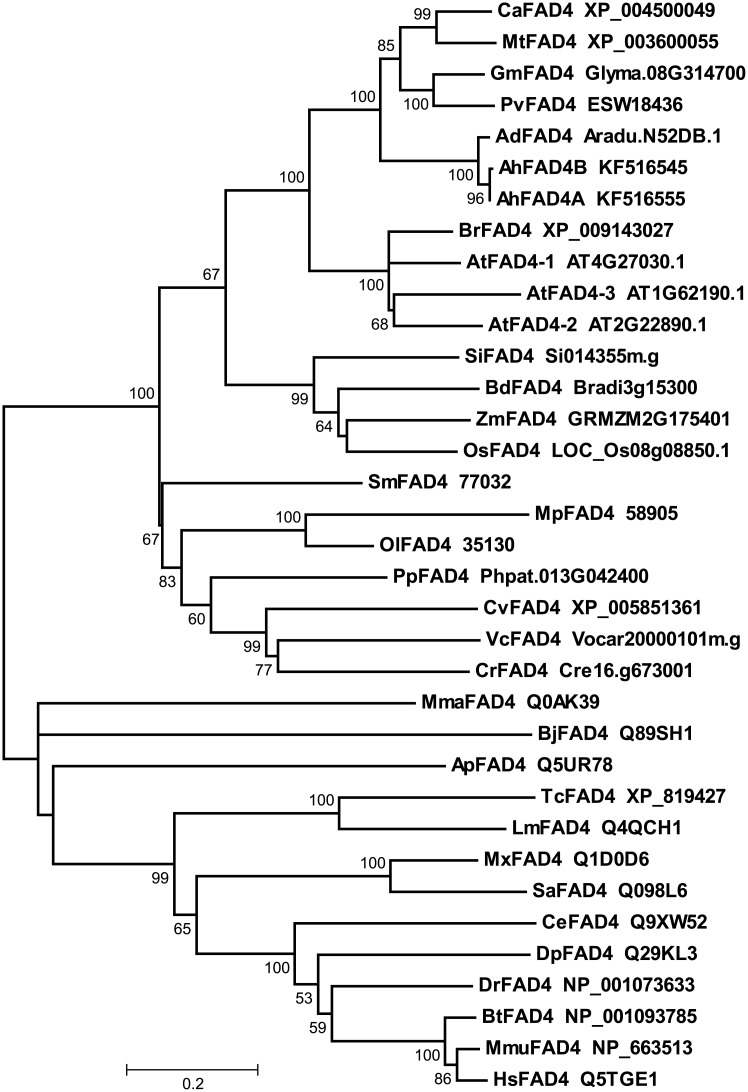
Phylogenetic tree of trans Δ3 desaturase gene families reconstructed by the neighbor-joining (NJ) method. Gene sequences were shown by their strain names, accession numbers (locus tags), or labels. Bootstrapping with 1,000 replicates was used to establish the confidence limits of the tree branches.

**Fig 3:**
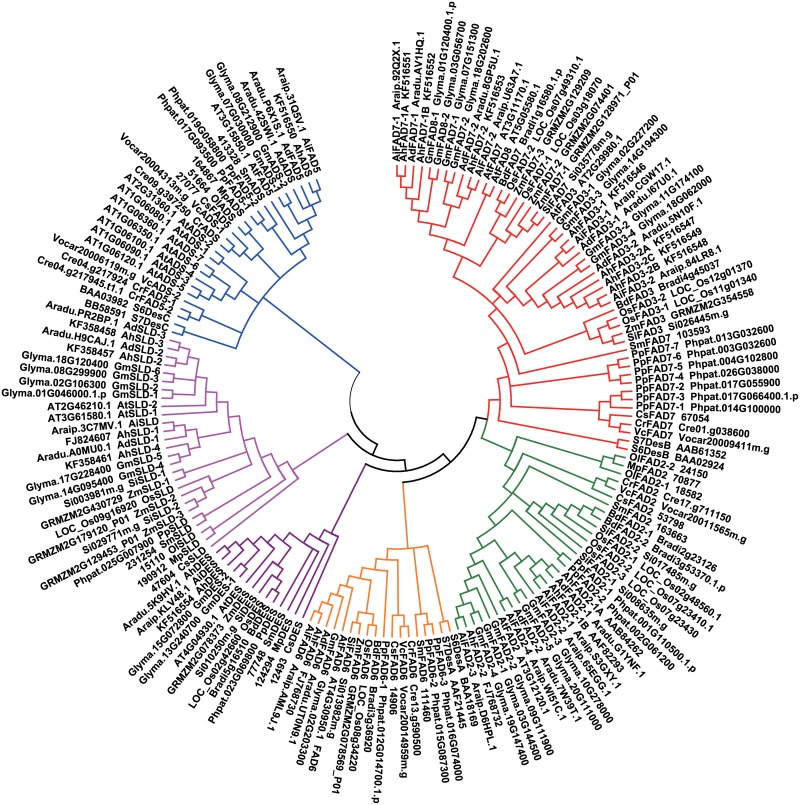
Phylogenetic tree of membrane desaturase gene families reconstructed by the neighbor-joining (NJ) method. Gene sequences were shown by their strain names, accession numbers (locus tags), or labels. Bootstrapping with 1,000 replicates was used to establish the confidence limits of the tree branches. Colored branches indicated different groups of proteins. Red: Δ15 desaturase, blue: Δ7/Δ9 desaturase, purple: sphingolipid Δ4 desaturase, green: microsomal Δ12 desaturase, orange: plastidial Δ12 desaturase, pink: sphingolipid Δ8 desaturase.

To examine the relationships among different sources of *FAD* genes, sequences from representative eukaryotic species belonging to plant monocotyledons (*Oryza sativa*, *Brachypodium distachyon*, *Setaria italica*, and *Zea may*), eudicots (*Arabidopsis thaliana*, *Glycine max*, *Arachis duranensis*, and *Arachis ipaensis*), a fern (*Selaginella moellendorfii*), a moss (*Physcomitrella patens*), and algae (*Chlamydomonas reinhardtii*, *Volvox carteri*, *Ostreococcus lucimarinus*, *Micromonas pusilla* RCC1545, and *Coccomyxa subellipsoidea* C-169), were selected to construct a phylogenetic tree by the neighbor-joining method (Figs 1–3). No homologues of any *ADS* genes were found in the genomes of rice (*Oryza sativa*), *Brachypodium distachyon*, *Setaria italica*, or maize (*Zea mays*), which suggested that *ADS* genes may be absent in monocots.

The polyunsaturated fatty acids are synthesized by two distinct pathways in plants, known as the prokaryotic and eukaryotic pathways, which are located within the membrane of plastids and the endoplasmic reticulum (ER), respectively [40]. Therefore, plant desaturases fall into two major classes: soluble and membrane-bound desaturases. The soluble desaturases are analyzed separately from membrane-bound desaturases because they are restricted to higher plants and show no evolutionary relationship with the more widely distributed membrane desaturases [39].

The plant stearoyl-ACP desaturase is the only soluble desaturase identified to date. In contrast, all other desaturases identified in plants, algae, animals, and fungi are integral membrane proteins [4, 41]. The phylogenetic tree indicated that three AhFAB2 genes were grouped with the stearoyl-ACP desaturases of higher plants and were distinct from those of the fern, the moss, and the green algae (Fig 1). This may suggest that stearoyl-ACP desaturases in the fern/moss/green algae and higher plants arose by independent gene duplication events. The three AhFAB2 genes were clustered into two subgroups. The AhFAB2-2 and AhFAB2-3 proteins were grouped together with FAB2 enzymes from eudicots and were separate from those of monocotyledons, while AhFAB2-1 and AtFAB2-1 clustered together with genes from eudicots and were set apart from the monocotyledon genes.

FAD4 encodes a predicted integral membrane protein that evolved independently from classic membrane bound desaturases, which were analyzed separately from the remaining membrane-bound desaturases [37]. Moreover this protein class, originally designated Kua proteins [42], is highly conserved in organisms ranging from bacteria (but not cyanobacteria) to mammals. Unfortunately, definitive functional data are not available for these proteins. The phylogenetic tree showed that the AhFAD4 protein was more closely related to enzymes from higher plants, the fern, the moss, and the green algae, and were separate from those of bacteria and animals (Fig 2).

As shown in the phylogenetic tree (Fig 3), all of the remaining membrane-bound desaturases fell into four distinct subfamilies: the Δ7/Δ9 desaturase subfamily, the Δ12/ω3 desaturase subfamily, the sphingolipid Δ8 desaturase subfamily, and the sphingolipid Δ4 desaturase subfamily. Δ9 desaturase is assumed to be the ancestor of the remaining desaturases based on functional criteria and the position of the clade integrated by Δ9 desaturases [43]. The AhADS gene was grouped with eudicot Δ7 homologs and was set apart from Δ7 enzymes in the fern, the moss, and the green algae, while the ADS genes in green algae and cyanobacteria were placed in a basal position.

In the Δ12/ω3 desaturase subfamily, the AhFAD6 gene, grouped to eudicot chloroplastic Δ12 desaturase, was located along with the cyanobacterial Δ12 desaturases at the basal position of the tree. The higher plant microsomal Δ12 desaturases formed a group and were set apart from those of the fern and the green algae. The AhFAD2-1A and AhFAD2-1B genes clustered together and were separate from the AhFAD2-2 gene. The cyanobacterial ω3 desaturases were placed in a basal position and were grouped with both microsomal and chloroplastic ω3 desaturases from higher plants, the fern, the moss, and the green algae. Seven putative ω3 desaturases from peanut (four FAD3 and three FAD7) were grouped with their respective microsomal or chloroplastic ω3 desaturases from higher plants and were separate from the fern, moss, and green algae enzymes. Therefore, it can be speculated that the cyanobacterial Δ12 desaturase might be the origin of plant Δ12 and ω3 desaturases, including both chloroplast and ER isozymes.

The sphingolipid Δ8 desaturases formed a separate clade (Fig 3). The SLD genes of green algae were placed in a basal position. The four peanut sphingolipid Δ8 desaturase genes (AhSLD-1, AhSLD-2, AhSLD-3, and AhSLD-4) clustered with SLDs from eudicots and were separate from those of monocotyledons. The sphingolipid Δ4 desaturases integrated into one clade (Fig 3). The AhDES gene, grouped with the eudicot DES genes, was located along with DES desaturases from green algae at the basal position of the tree.

### Tissue-specific expression patterns

**Fig 4:**
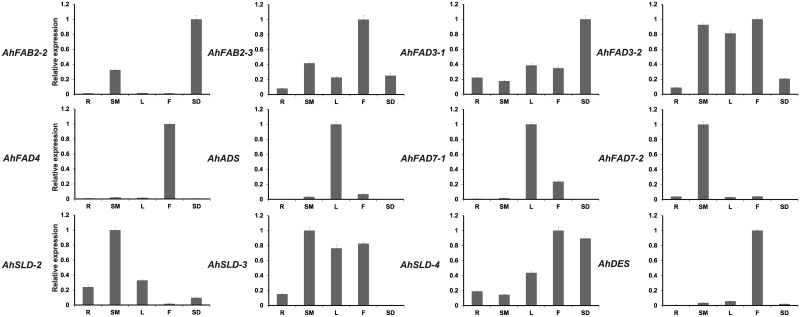
Expression analysis of fatty acid desaturase genes of peanut using qRT-PCR in five peanut tissues. R, root; SM, stem; L, leaf; F, flower; SD, seed. The relative mRNA abundance was normalized with respect to the peanut *AhTUA5* gene. The bars were standard deviations (SD) of three biological repeats.

**Fig 5:**
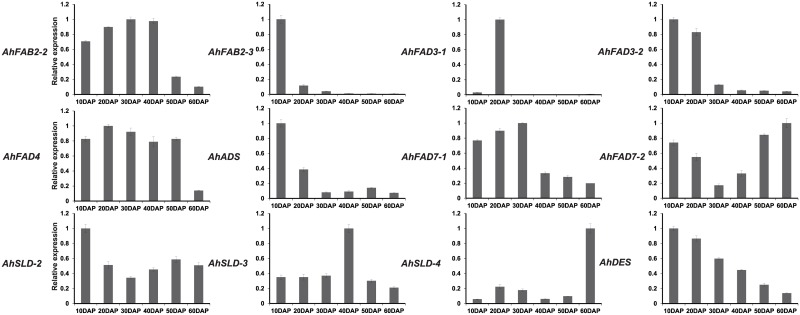
Expression analysis of fatty acid desaturase genes of peanut using qRT-PCR at six stages of seed development. DS (10 to 60 DAP): six developmental stages of seeds. The relative mRNA abundance was normalized with respect to the peanut *AhTUA5* gene. The bars were standard deviations (SD) of three biological repeats.

Quantitative real-time PCR (qRT-PCR) was used to confirm the expression patterns of the seventeen *FAD* genes in different peanut tissues and at different stages of seed development. The actin 11 (*AhACT11*) gene was used as an internal reference control for total RNA input [44]. Figs 4 and 5 shows that these genes displayed specific temporal and spatial expression patterns across different tissues and developmental stages. *AhFAB2-2* and *AhFAD3-1* transcripts were more abundant in seeds than in any of the other tissues tested. The highest transcript accumulations of *AhFAB2-3*, *AhFAD3-2*, *AhFAD4*, *AhSLD-4*, and *AhDES* genes occurred in flowers. Among them, *AhFAB2-3* and *AhFAD3-2* had relatively higher expressions in stems. *AhSLD-4* was expressed most strongly in flowers followed by seeds, whereas the expressions of the *AhFAD4* and *AhDES* gene were largely restricted to flowers. *AhADS* and *AhFAD7-1* had similar expression patterns, and were most abundant in leaves followed by flowers. The highest abundances of *AhFAD7-2* and *AhSLD-2* transcripts were in stems. *AhSLD-3* transcript levels were highest in stems, followed by flowers, leaves, and roots, with the lowest levels being found in seeds.

The expression patterns of *FAD* genes across six seed developmental stages are shown in Fig 5. The *AhSLD-3* transcript levels remained relatively low during the initial stage of seed development, but increased gradually during the later stages, peaking at 40 DAP, and decreased thereafter until 60 DAP. The expressions of *AhFAB2-2 and AhFAD7-1* gradually increased in abundance during seed development, reached a maximum expression level at 30 DAP, and then decreased thereafter. The *AhFAB2-3*, *AhFAD3-2*, and *AhADS* transcript levels were highest at 10 DAP and decreased dramatically thereafter. The *AhSLD-2* transcript level remained relatively high at the initial stage of seed development, but decreased gradually from 10 to 20 DAP, and then increased from 30 DAP. The *AhFAD3-1* gene showed higher expression levels at 20 DAPs and much lower levels during the other stages. The expression levels of *AhDES* were highest at the initial stage of seed development, but gradually decreased in abundance during the later stages. The *AhSLD-4* transcript levels remained relatively low at the initial stage of seed development, but showed a dramatic increase in abundance at 60 DAP. The *AhFAD4* transcript levels remained relatively high in the initial five stages, but showed a dramatic decrease thereafter. The *AhFAD7-2* transcript level remained relatively high at the initial stage of seed development, decreased gradually from 10 to 30 DAP, and then increased from 40 DAP, with the highest expression being seen at 60 DAP.

Many desaturase genes from different plant species have been studied and their expression levels are regulated in a tissue-specific manner. Our results indicated that the expression of the *AhDES* gene was largely restricted to flowers, which highly resembled the expression pattern of its closest ortholog *AtDES* in *Arabidopsis* with preferential expressions in pollen and floral tissues [45]. It has been reported that *AtFAD2* (AT3G12120) and *AtFAD6* (AT4G30950) mRNAs are present in various *Arabidopsis* tissues, including the roots, rosette leaves, cauline leaves, stems, flowers, and siliques [46, 47]. The *AhFAD2-1*, *AhFAD2-2*, and *AhFAD6* genes were expressed in all tissues surveyed, which was consistent with the *Arabidopsis* orthologs [17]. In *Arabidopsis*, *AtSLD-1* (At3g61580) was ubiquitously expressed in all organs and most highly expressed in the flowers. However, *AtSLD-2* (At2g46210) was expressed in flowers and siliques, but shows only low levels of expression in the leaves, stems, and roots [48]. The *AhSLD-4* gene transcript accumulated to the greatest extent in flowers, which was consistent with the *Arabidopsis* orthologs. The *AhSLD-1* transcript was more abundant in leaves, whereas *AhSLD-2* and *AhSLD-3* were most strongly expressed in stems. In *Arabidopsis*, *AtFAB2-1* (At2g43710) and *AtFAB2-7* (At5g16240) were expressed at high levels in the flower, stem and leaf tissues, but at low levels in roots and siliques. *AtFAB2-4* (At3g02620) was relatively highly expressed in roots compared to its levels in the leaf, stem, flower and silique tissues, while *AtFAB2-6* (At5g16230) was highly expressed in the leaves, but not in the roots. The *AtFAB2-5* (At3g02630) isoform was expressed at high levels in the leaf, stem and flower tissues, but at low levels in roots and siliques. The *AtFAB2-3* (At3g02610) isoform was only expressed at low levels in the roots and flowers, while *AtFAB2-2* (At1g43800) was not detected in any of the tissues analyzed [49]. In peanut, *AhFAB2-1* and *AhFAB2-2* showed higher transcript abundances in seeds than in any of the other tissues tested [17], whereas the *AhFAB2-3* gene accumulated in flowers. In *Arabidopsis*, the *AtADS-1* (At1g06080) gene was expressed most strongly in inflorescence meristems followed by leaves and flowers, and very weakly in roots and seedpods. The *AtADS-2* (At2g31360) gene was expressed strongly in all the analyzed organs, although the expression level was higher in flowers and roots than in other organs [50, 51]. In peanut, *AhADS* transcript levels were higher in the leaves, followed by flowers, and the lowest levels occurred in the seeds and roots. The *AtFAD3* gene was expressed in both the leaves and roots, while the *AtFAD7* gene transcript was only observed in the photosynthetically active organs of *Arabidopsis* [52]. In peanut, the *AhFAD3-1* transcript abundances were higher in seeds than in the other tissues examined, whereas the *AhFAD3-2* transcripts were more abundant in flowers. The *AhFAD7-1* gene transcript accumulation was highest in leaves, whereas *AhFAD7-2* was expressed most strongly in stems. In *Gossypium raimondii*, *GrFAD3*.*2*, *GrFAD8*.*1*, and *GrSLD5* were expressed at high levels in roots. *GrFAD2*.*3*, *GrSLD3*, *GrSLD2*, *GrSLD1*, *GrFAD3*.*1*, and *GrDSD1* shared high expression levels in young stems. *GrFAD5* and *GrFAD6* displayed the highest transcript abundance in cotyledons. And *GrFAD7* and *GrFAD2*.*4* were predominantly expressed in leaves [53]. In Cucumber, three *CsFAB2* genes were dominantly expressed in the seedling leaves. For *CsFAD2*.*1*, *CsFAD3* and *CsFAD6*, the highest expression levels were detected in the leaves, whereas for *CsFAD4*, *CsFAD5*.*1* and *CsFAD7*, the highest transcript abundances were detected in the cotyledons. In the roots and hypocotyls, only trace expression levels could be detected for any cucumber *FAD* gene except *CsFAD2*.*1* and *CsFAD3* [54]. In *Perilla frutescens*, *PfrFAD2* and *PfrFAD3* genes were expressed in leaves and during all stages of seed development, and their expression levels in 2- to 3-week-old developing seeds were 6.7- and 25-fold higher than their expression in leaves, respectively. In contrast, although the expression of *PfrFAD7-1* and *PfrFAD7-2* was similar to that of *PfrFAD2* and *PfrFAD3* genes in leaves, their expressions were much lower in developing seeds [55]. Thus, the same type of *FAD* genes from different plants may have different spatial expression patterns, which requires further investigation.

### Expression patterns of *AhFADs* in peanut under abiotic stress

**Fig 6:**
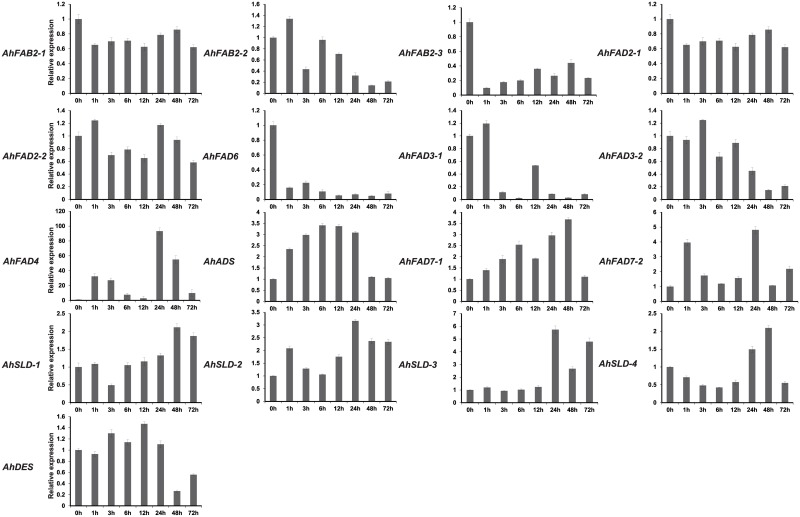
Expression analysis of fatty acid desaturase genes of peanut using qRT-PCR in peanut leaves upon cold treatment. 0h to 72h, leaves exposed to cold (4°C) treatment. The relative mRNA abundance was normalized with respect to the peanut *AhTUA5* gene. The bars were standard deviations (SD) of three biological repeats.

We monitored changes to these transcripts in peanut leaves and roots to confirm the expression patterns of these *FAD* genes, including five previously cloned desaturase genes, under cold, salt, drought, and ABA stresses. Two microsomal oleoyl-PC desaturase genes (*AhFAD2-1A* and *AhFAD2-1B*) have been identified in peanut and their open reading frames (ORFs) were 99% identical [19, 20]. Thus, the gene-specific primers used for the amplification of *AhFAD2-1* in our analysis recognized and amplified both *AhFAD2-1A* and *AhFAD2-1B* genes. Fig 6 shows the expression patterns of these *FAD* genes in peanut leaves after cold treatment. There were no obvious changes in the abundances of the *AhFAB2-1*, *AhFAB2-2*, *AhFAB2-3*, *AhFAD2-1*, *AhFAD2-2*, *AhFAD6*, *AhFAD3-1*, *AhFAD3-2*, and *AhDES* transcripts in peanut leaves after cold treatment. The expressions of *AhFAD4*, *AhFAD7-2*, and *AhSLD-2* increased slightly at 1 h after treatment, and then decreased from 3 h to 12 h. After 24 h, their transcript levels reached a maximum, which were approximately 93-, 5- and 3-fold, higher than the non-treated controls, respectively. The expression levels of *AhADS* increased under cold stress, peaking at 6 h, and then decreased. The greatest increase was about 3.6-fold. The expression of *AhSLD-1* increased slightly after 1 h treatment with cold and then decreased. After 48 h, expression of the *AhSLD-1* transcript reached its maximum level, with a nearly 2-fold increase. The transcript levels of *AhFAD7-1*, *AhSLD-3*, and *AhSLD-4* gradually increased under cold stress, peaking at 48 h, 24 h, and 48 h, with approximately 4-, 6- and 2-fold increases, respectively, compared to the non-treated controls.

**Fig 7:**
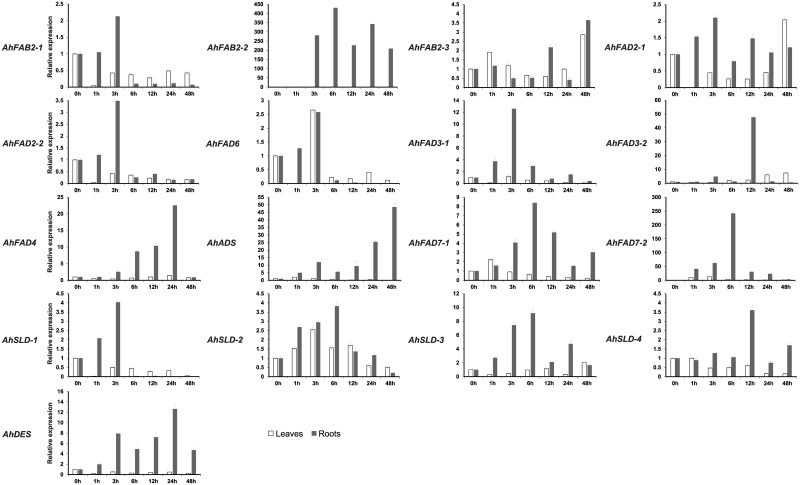
Expression analysis of fatty acid desaturase genes of peanut using qRT-PCR in peanut leaves and roots under salt stress. 0h to 48h, leaves exposed to high salt (200 mM NaCl) treatment. The relative mRNA abundance was normalized with respect to the peanut *AhTUA5* gene. The bars were standard deviations (SD) of three biological repeats.

The expression patterns of *AhFADs* in peanut leaves and roots after treatment with 200 mM NaCl were also monitored (Fig 7 and S4 Fig). In leaves, there were no obvious changes in the abundances of the *AhFAB2-1*, *AhFAB2-2*, *AhFAD2-2*, *AhFAD3-1*, *AhFAD4*, *AhSLD-1*, *AhSLD-4*, and *AhDES* transcripts after salt treatment. The transcript levels of *AhFAD6*, *AhADS*, *AhFAD7-1*, *AhFAD7-2*, and *AhSLD-2* gradually increased under salt stress, peaking at 3 h, 1 h, 1 h, 3 h, and 3 h, respectively, and showed approximately 3-, 2-, 2-, 13-, and 3-fold increases, respectively. The expressions of *AhFAD2-1* and *AhFAD3-2* gradually increased under salt stress, with peak levels that were about 2- and 7-fold higher, respectively, at 48 h. The expression of *AhFAB2-3* increased slightly at 1 h after treatment, and then decreased from 3 h to 12 h. After 48 h, *AhFAB2-3* transcripts reached a maximum level, with the greatest increase being approximately 3-fold. The expression of *AhSLD-3* slightly increased in the leaves of seedlings subjected to salt stress, with about a 2-fold peak increase at 48 h.

In roots, the levels of the *AhFAB2-1*, *AhFAD2-1*, *AhFAD2-2*, *AhFAD6*, *AhFAD3-1*, and *AhSLD-1* transcripts increased, and reached maximum levels at 3 h after salt treatment, with the greatest increases observed being about 2-, 2-, 3-, 3-, 13-, and 4-fold, respectively, compared to the non-treated controls. The expressions of *AhFAB2-2*, *AhFAD7-1*, *AhFAD7-2*, *AhSLD-2*, and *AhSLD-3* increased under salt stress, with a peak level at 6 h in roots, where the greatest increases were about 431-, 8-, 242-, 4-, and 9-fold, respectively, compared to the non-treated controls. The expression levels of *AhFAD3-2*, *AhSLD-4*, *AhFAD4*, and *AhDES* increased after salt treatment, peaking at 12 h or 24 h, with increases of approximately 48-, 4-, 23-, and 13-fold, respectively. The transcript levels of *AhADS* increased in roots under salt stress, with peak expression levels that were 49-fold greater at 48 h compared to the non-treated controls. The expression of *AhFAB2-3* increased gradually from 1 to 12 h after salt treatment and then decreased. After 48 h, expression of the *AhFAB2-3* transcript reached its maximum level, with a near 4-fold increase compared to the non-treated controls.

**Fig 8:**
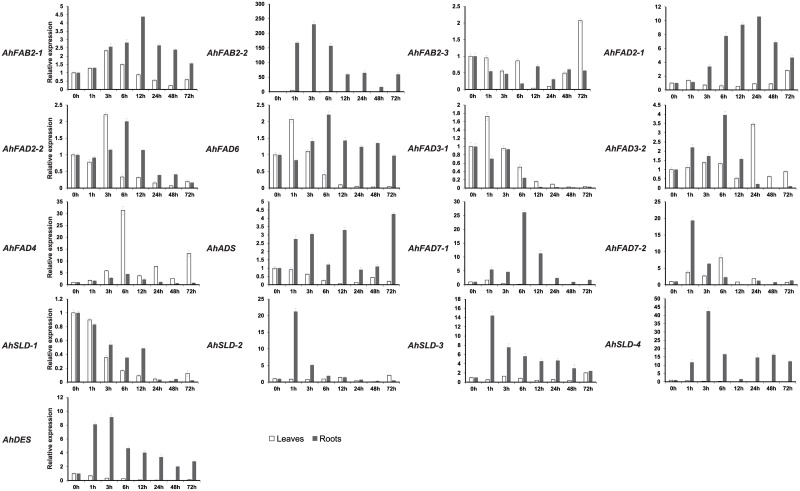
Expression analysis of fatty acid desaturase genes of peanut using qRT-PCR in peanut leaves and roots under drought stress. 0h to 72h, leaves exposed to 20% PEG-6000 treatment. The relative mRNA abundance was normalized with respect to the peanut *AhTUA5* gene. The bars were standard deviations (SD) of three biological repeats.

A 20% solution of PEG-6000 was used to mimic drought stress to monitor the expression patterns of *AhFADs* in peanut leaves and roots (Fig 8 and S4 Fig). There were no obvious changes in the abundances of the *AhFAD3-1*, *AhFAD7-1*, *AhSLD-1*, *AhADS*, *AhSLD-4*, and *AhDES* transcripts in peanut leaves after drought treatment. In leaves, the transcript levels of *AhFAB2-2*, *AhFAD6*, *AhFAD2-2*, *AhFAB2-1*, *AhFAD7-2*, and *AhFAD3-2* gradually increased under salt stress, peaking at 1 h, 1 h, 3 h, 6 h, 6 h, and 24 h, respectively, with increases of approximately 5-, 2-, 2-, 2-, 8-, and 3-fold, respectively, compared to the non-treated controls. The expressions of *AhFAB2-3*, *AhFAD2-1*, and *AhSLD-3* increased in the leaves of seedlings subjected to drought stress, with peak level increases of about 2-, 3-, and 2-fold, respectively, at 72 h. The expression of *AhFAD4* increased under drought stress, with a maximum increase of about 31-fold observed at 6 h, and then decreased from 12 h to 48 h. At 72 h, the *AhFAD4* transcript levels increased again. The expression of *AhSLD-2* increased gradually from 1 to 12 h after drought treatment and then decreased. After 72 h, expression of the *AhSLD-2* transcript reached a maximum level, with a nearly 2-fold increase compared to the non-treated controls.

In roots, there were no obvious changes in the abundances of the *AhFAB2-3*, *AhFAD3-1*, *and AhSLD-1* transcripts after drought treatment. The levels of *AhFAD2-2*, *AhFAD6*, *AhFAD3-2*, *AhFAD4*, and *AhFAD7-1* transcripts increased, and reached their maximum levels 6 h after drought treatment, with the greatest increases observed being about 2-, 2-, 4-, 4-, and 26-fold higher, respectively, than the non-treated controls. The transcript levels of *AhFAD7-2*, *AhSLD-2*, *AhSLD-3*, *AhFAB2-2*, *AhDES*, *AhFAB2-1*, and *AhFAD2-1* gradually increased under salt stress, peaking at 1 h, 1 h, 1 h, 3 h, 3 h, 12 h, and 24 h, with increases of approximately 19-, 21-, 14-, 231-, 9-, 4-, and 11-fold, respectively, compared to the non-treated controls. The expression of *AhADS* gradually increased under drought stress, with a maximum increase of about 4-fold observed at 72 h. The expression of *AhSLD-4* increased under drought stress, with a maximum increase of about 43-fold observed at 3 h, and then decreased from 6 h to 12 h. After 24 h, the *AhSLD-4* transcript levels increased again.

**Fig 9:**
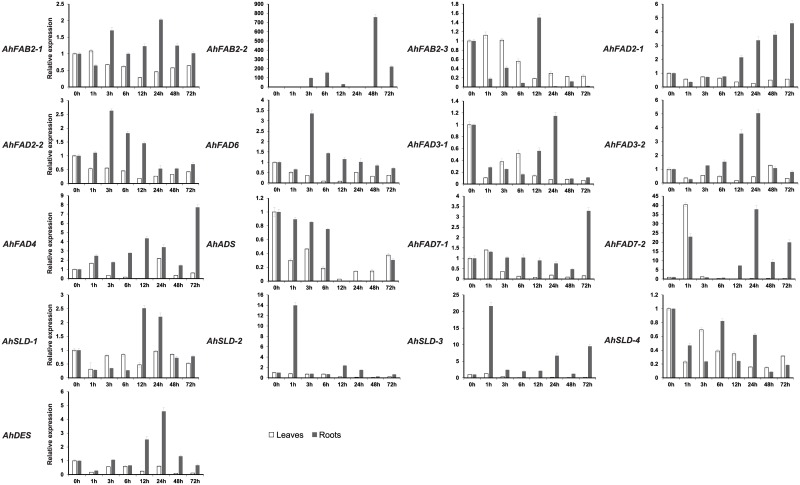
Expression analysis of fatty acid desaturase genes of peanut using qRT-PCR in peanut leaves and roots under ABA treatment. 0h to 72h, leaves exposed to 100uM ABA treatment. The relative mRNA abundance was normalized with respect to the peanut *AhTUA5* gene. The bars were standard deviations (SD) of three biological repeats.

We also examined the response of *AhFAD* genes to exogenously applied ABA, which is a plant signaling molecule involved in plant defense signaling pathways (Fig 9 and S4 Fig). In leaves, there were no obvious changes in the abundances of the *AhFAB2-1*, *AhFAB2-3*, *AhFAD2-1*, *AhFAD2-2*, *AhFAD3-1*, *AhFAD3-2*, *AhFAD6*, *AhADS*, *AhFAD7-1*, *AhSLD-1*, *AhSLD-2*, *AhSLD-3*, *AhSLD-4*, and *AhDES* transcripts after ABA treatment. The transcript levels of *AhFAD7-2*, and *AhFAB2-2* increased after ABA treatment, peaking at 1 h and 48 h, respectively, with increases of approximately 40- and 2- fold compared to the non-treated controls. The expression of *AhFAD4* increased rapidly 1 h after treatment, and then decreased from 3 h to 12 h. After 24 h, *AhFAD4* transcripts reached a maximum level, with an approximately 2-fold increase.

In roots, there were no obvious changes in the abundances of the *AhFAB2-3*, *AhFAD3-1*, *AhADS*, and *AhSLD-4* transcripts after ABA treatment. The transcript levels of *AhSLD-2*, *AhSLD-3*, *AhFAD2-2*, *AhFAD6*, *AhSLD-1*, *AhFAD3-2*, and *AhDES* gradually increased after ABA treatment, peaking at 1 h, 1 h, 3 h, 3 h, 12 h, 24 h, and 24 h, respectively, with increases of approximately 13-, 22-, 3-, 3-, 3-, 5-, and 5-fold compared to the non-treated controls. The *AhFAD2-1*, *AhFAD7-1*, and *AhFAD4* transcripts levels were considerably higher in ABA-treated roots than in untreated roots after 72 h, with a maximum increase of approximately 5-, 3-, and 8-fold, respectively. The *AhFAD7-2* and *AhFAB2-1* expressions increased after 1 h or 3 h treatment with ABA and then decreased. After 24 h, the *AhFAD7-2* and *AhFAB2-1* transcript levels reached a maximum, with the greatest increase being approximately 37- and 2-fold, respectively. The *AhFAB2-2* transcript levels increased after 6 h treatment, but decreased from 12 h to 24 h. After 48 h, the expression of *AhFAB2-2* increased with a peak level of 757-fold.

Environmental factors compel organisms to acclimatize to the external conditions [56]. Poikilothermic organisms, such as cyanobacteria and plants, modulate the composition of their membrane lipids in response to changes in environmental conditions [57, 58]. Unsaturated fatty acids are essential constituents of glycerolipids in biological membranes and the unsaturation level of membrane lipids is important in controlling the fluidity of membranes [59]. The extent of unsaturation is mainly determined by the activity of fatty acid desaturases, the enzymes that introduce double bonds into specific positions of lipid fatty-acyl chains [60]. Previous studies have revealed that *FAD* genes are crucial for the survival of plants faced with different environmental stresses. In *Arabidopsis*, the expression of *AtFAD8* was strongly induced by cold temperatures [9]. *AtFAD2* and *AtFAD6* were found to be active in seedlings under salinity stress [46, 47]. The *ads2* mutant *Arabidopsis* plants showed increased sensitivity to chilling and freezing temperatures [61], and the mutants of *AtSLD* genes also showed enhanced sensitivity to prolonged low-temperature exposure [48]. In transgenic tobacco plants, over-expressing *Arabidopsis AtFAD7* also enhanced cold tolerance [62], whereas antisense expression of *AtFAD7* reduced salt and drought tolerance [63]. Tobacco plants overexpressing either *Arabidopsis AtFAD3* or *AtFAD8* gene also exhibited increased tolerance to drought and osmotic stress [64]. In *Saussurea involucrate*, the expression of *sikSACPD* increased in leaves as the temperature decreased from 20 to -10°C. The *FAB2*:*SikSACPD* transgenic plants showed a slightly more resistance to the freezing stress than the *FAB2*:*FAB2* transgenic plants and the wild-type [65]. In *Gossypium raimondii*, *GrFAD8*.*1*, *GrFAD2*.*2*, *GrFAD8*.*2*, *GrSLD2*, *GrSLD4*, *GrDSD1* and *GrSLD5* were found to be significantly up-regulated in response to cold stress. Conversely, *GrFAD5*, *GrFAD7*, *GrFAD2*.*3*, *GrSLD1* and *GrSLD3* were heavily down-regulated after long periods of cold stress treatment [53]. In safflower, the transcription level of *CtFAD3* remained constant at all different growth temperatures in the leaves; in contrast, the accumulation of *CtFAD7* mRNA slightly increased at low temperature, while *CtFAD8* mRNA decreased significantly. The expressions of *CtFAD3*, *CtFAD7*, and *CtFAD8* in the roots significantly increased at low temperature [66]. In rice, *OsFAD8* has been reported to have a functional role in stress tolerance at low temperatures [67]. In tomato, *LeFAD3* overexpression enhanced the tolerance of tomato seedlings for salinity stress [68], whereas silencing the *LeFAD7* gene alleviated high-temperature stress [69]. In soybean, the expression of *FAD3* and *FAD7* was tightly regulated in response to cold temperature [70].

Our results indicated that *FAD* transcripts from peanut were differentially expressed following exposure to abiotic stresses or a stress-induced plant hormone. The *AhFAD7-2* transcript levels were considerably enhanced under all stress treatments. The expressions of *AhFAD4*, *AhSLD-2*, and *AhSLD-3* increased in all materials after the stress treatments, except for salt- or ABA-treated leaves, whereas the transcript levels of *AhFAD3-1* only increased in salt-stressed roots. The *AhFAB2-2*, *AhFAD2-1*, *AhFAD6*, and *AhFAD2-3* transcript levels were distinctly enhanced after exposure to four kinds of stress separately, except for cold-, salt-treated leaves or cold-, ABA-treated leaves. The expressions of *AhFAD7-1* increased in all materials after the stress treatments, except for drought- and ABA-treated leaves. The transcript levels of *AhFAB2-1* and *AhFAD2-2* increased in salt-, drought-, and ABA-treated roots, and drought-treated leaves, whereas the expression of *AhADS* increased in cold-, salt-treated leaves and salt-, drought-treated roots. The *AhDES* transcripts levels increased substantially in roots exposed to salt, drought, and ABA stresses, whereas the *AhSLD-4* transcript levels were distinctly enhanced in salt-, drought-treated roots and cold-treated leaves. *AhSLD-1* expression increased in salt- or ABA-treated roots and cold-treated leaves, whereas *AhFAB2-3* transcript levels increased in salt-, drought-stressed leaves and salt-treated roots. Taken together, these results from qRT-PCR suggested that the expression of most peanut *FAD* genes was induced by stress treatment, consistent with the *FAD* genes from other plants [61–70]. To comprehensively decipher their functions involved in stress tolerance in peanut seedlings, some lipidomic and transcriptomic methods would be employed.

### Heterologous expression of *AhFAD6* in *Synechococcus elongatus* (strain PCC 7942)

*Synechococcus elongatus* (strain PCC 7942) is a freshwater unicellular cyanobacterium that only contains monounsaturated fatty acids. It is an excellent model system for studying fatty acid metabolism. When the Δ12 fatty acid desaturase gene is introduced into the genome of *S*. *elongates*, the resultant cells will produce considerable amounts of diunsaturated fatty acids.

Heterologous expression in *S*. *elongates* was used to confirm Δ12 regioselectivity and the function of *AhFAD6* genes. The pYFAD6 and empty vector (pSyn_1, control) were transformed into the *S*. *elongates*. The total lipids of the transformants were analyzed using GC-MS. The results showed a novel fatty acid peak from pYFAD6, which was absent in the control. The novel fatty acid was designated as C18:2 by comparing the retention time to FAME standard mixtures (Sigma). No C16:2 was detected, which indicated that *AhFAD6* recognized only one substrate (C18:1) in *S*. *elongates* (Table 2). The C18:2 percentage was 3.3% for pYFAD6 transformants. These data showed that the activity of the *AhFAD6* protein was significantly higher in the cyanobacterium than in yeast, where the percentage of C18:2 was 0.1% [17]. Yeast is known to be the model of choice for the functional characterization of microsomal FADs because it contains the short electron transport system required by these desaturases (i.e., cytochrome b5 and NADH-cytochrome b5 reductase) [71]. Nevertheless, the high desaturation level evident from Table 2 suggested that desaturases of plastidial origin, which usually require ferredoxin and NADPH-ferredoxin reductase, were supplied to some extent with reducing equivalents in yeast cells.

**Table 2:** Fatty acid composition of transformed *Synechococcus elongatus* (strain PCC 7942).

Transformant	Percent of total fatty acids					
	16:0	16:1	16:2	18:0	18:1	18:2
pSyn_1	49.48	30.03	-^a^	0.45	20.05	-
pYFAD6	44.05	28.56	-^a^	2.88	21.20	3.30

^a^ Dashes indicated that the fatty acid was not detected.

It is well known that the *AhFAD2-1* gene plays a major role in the conversion of oleic to linoleic acid in seed storage oils [19, 20, 72]. Two other genes, *AhFAD2-2* and *AhFAD6* have been isolated by us [17]. They also contribute to the C18:2 pool, although a major portion of this pool reflects contributions from AhFAD2-1 activity. This may indicated that a switch from oleic acid to linoleic acid might involve more Δ12 desaturase genes and an intricate metabolic network that regulates linoleic acid biosynthesis between the endoplasmic reticulum and the chloroplast within peanut cells. The functional validation of these two novel members would facilitate the further genetic manipulation of the peanut oil quality trait that is based on a high O/L ratio [18].

### Heterologous expression of *AhSLDs* in *Saccharomyces cerevisiae*

**Fig 10:**
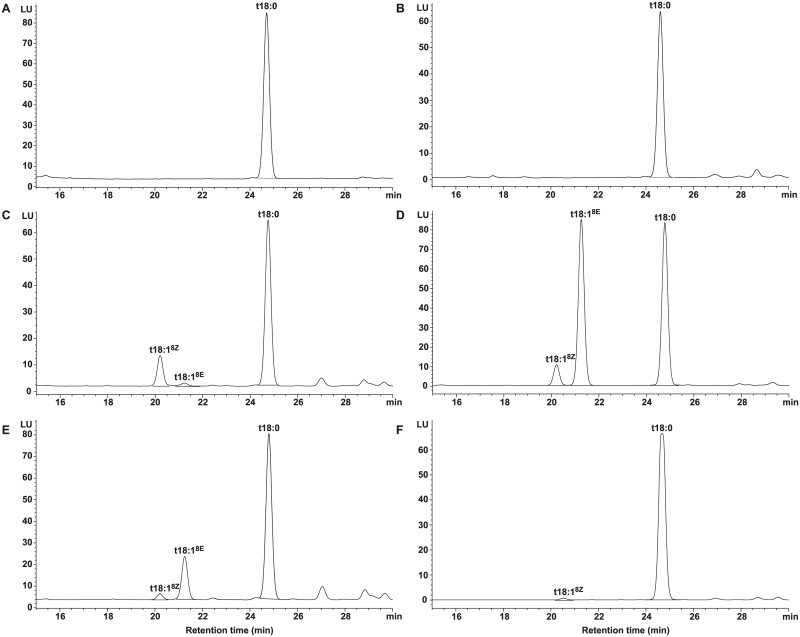
Formation of phytosphingenines in yeast cells by heterologous expression of sphingolipid desaturase from peanut. A: t18:0, used as the standard. B: the predominating LCBs from *S*. *cerevisiae* cells (INVSc1) harbouring the empty vector pYES2 with t18:0 as the control. C-F: formation of t18:1^8Z^ and t18:1^8E^ in yeast cells expressing pYES2-AhSLD-1/2/3/4. LCBs from yeast cells were converted into their fluorescent derivatives and analyzed by RP-HPLC.

In order to elucidate whether *AhSLD-1*, *AhSLD-2*, *AhSLD-3*, and *AhSLD-4* encode functional Δ8 sphingolipid desaturases, these four genes were expressed in *S*. *cerevisiae* under the control of the galactose-inducible GAL1 promoter. Reverse-phase high-performance liquid chromatography (RP-HPLC) showed that when transformed with the empty vector (control), yeast cells showed an LCB pattern that was identical to the wild-type pattern, which contained mainly C18-phytosphinganine (t18:0) (Fig 10). In contrast, yeast transformants containing *AhSLD-1*, *AhSLD-2*, *AhSLD-3*, and *AhSLD-4* accumulated novel (Z)- and (E)-desaturated sphingoid bases with productions of 17.63%, 53.5%, 23.4%, and 0.92%, respectively, in addition to t18:0 (Fig 10 and Table 3). The ratio of the newly synthesized 8(Z)-C18-phytosphinganine (t18:1^8Z^) and 8(E)-C18-phytosphinganine (t18:1^8E^) was quite different in the four transformants: the ratio for *AhSLD-1* was 7.74, and those for *AhSLD-2* and *AhSLD-3* were 0.13. *AhSLD-4* only newly synthesized t18:1^8Z^. These results indicated that *AhSLD-1*, *AhSLD-2*, *AhSLD-3*, and *AhSLD-4* all encoded functional Δ8 sphingolipid desaturases with diverse biochemical functions. In a similar way, four *BrSLD1* genes in *Brassica rapa* have been isolated, which also catalyze different ratios of t18:1^8Z^ and t18:1^8E^ [73]. However, two different genes encoding sphingolipid Δ8 desaturase were discovered in *Arabidopsis* and *Helianthus annuus*, there have been no reports on different product ratios in these two species [48, 74, 75]. Our study will help further elucidate the key domains determining t18:1^8Z^ and t18:1^8E^ biosynthesis using domain swapping between these similar enzymes.

**Table 3:** The ratios of t18:1^8Z^ and t18:1^8E^ and the conversion rate of the four *SLD* genes of peanut.

Gene name	t18:1^8Z^/t18:1^8E^	Conversion rate (%)^a^
*AhSLD-1*	7.74	17.63
*AhSLD-2*	0.13	53.5
*AhSLD-3*	0.13	23.4
*AhSLD-4*	-^b^	0.92

^a^Conversion rate (%) = (t18:1^8Z^ + t18:1^8E^)/(t18:1^8Z^+t18:1^8E^+t18:0)×100.

^b^ Dashes indicated that the t18:1^8E^ was not detected.

## Conclusions

In conclusion, twelve novel *FAD*-like genes from peanut were cloned, including two *FAB2*, two *FAD3*, two *FAD7*, one *FAD4*, one *ADS*, one *DES*, and three *SLD* genes. The functions of one *AhFAD6* and four *AhSLD* genes were verified by heterologous expression in *S*. *elongates* or *S*. *cerevisiae*. Better understanding of this enzyme family will improve efforts to modify the content and composition of seed oils or improve abiotic stress resistance in plants. The results generated in our study provide new information that will increase our understanding of the evolution, functional diversity and gene expression of fatty acid desaturases in plants and opens the way to select candidate genes for functional validation studies in peanut.

## Materials and methods

### Ethics statement

No specific permits were required for the described field studies. No specific permissions were required for these locations and activities. The location is not privately-owned or protected in any way and the field studies did not involve endangered or protected species.

### Plant materials

Peanut plants (*A*. *hypogaea* L. cultivar Huayu 19) were grown in a growth chamber with a 16 h light/8 h dark photoperiod at 26°C/22°C day/night temperatures. Leaves, stems and roots were sampled from the seedlings at the trefoil leaf stage. Seeds were sampled at 10, 20, 30, 40, 50, and 60 days after pegging (DAP). Flowers were collected when the seedlings were in the flowering phase. For the cold treatment, seedlings in the soil at the trefoil leaf stage were kept at 4°C, and leaves were sampled separately either before cold treatment (0 h) or after continuous exposure to 4°C for 1, 3, 6, 12, 24, 48, or 72 h. For stress treatments, roots of seedlings grown in soil were flushed carefully with tap water to remove all soil, and then submerged in solutions of 200 mM NaCl, 20% PEG-6000, or 100 μM ABA. Leaves and roots were sampled separately after treatment for 0, 1, 3, 6, 12, 24, 48, or 72 h. All samples were immediately frozen in liquid nitrogen and stored at -80°C until required.

### Identification of *FAD* family genes in a peanut cDNA library using Bioedit software

The amino acid sequences of *FAD* genes of *Arabidopsis*, *AtFAB2-1* (AT2G43710), *AtADS-1* (AT1G06080), *AtFAD2* (AT3G12120), *AtFAD4-1* (AT4G27030), *AtFAD6* (AT4G30950), *AtFAD7* (AT3G11170), *AtFAD8* (AT5G05580), *AtFAD3* (AT2G29980), *AtFAD5* (AT3G15850), and *AtDES* (AT4G04930), were used as query to search for homologous genes from the peanut cDNA library including 36,741 cDNA sequences. Before searching for members of the *FAD* gene family, a local nucleotide database file was created using Bioedit software [35]. A local BLAST procedure was then run to find the homologous genes of the *FAD* family. Using this method, we found twelve genes that may encode *FAD* proteins.

### Isolation of full-length cDNA sequences

Total RNA was extracted using the RNeasy Plant Mini kit (Qiagen, Valencia, CA, USA). Contamination with genomic DNA was eliminated by treatment with recombinant DNase I (Qiagen), as recommended by the vendor. Only RNA preparations having an A260/A280 ratio of 1.8–2.0 and an A260/A230 ratio >2.0 were used for subsequent analysis. The integrity of RNA was verified by electrophoresis through 2% agarose gels, followed by SYBR Green staining. First-strand cDNA synthesis was carried out with 2 μg RNA using an RT-PCR kit (Promega, Madison, WI, USA) according to the manufacturer’s procedure.

We performed PCR with the LA PCR system (Takara, Dalian, China), using 2.5 μl of 10×PCR buffer with MgCl_2_, 1 μl of each primer (10 μM) (S2 Table), 4.0 μl of 10 mM dNTPs, 1 μl of cDNA sample, 0.5 μl of LA Taq^™^ DNA polymerase, and 15 μl of double-distilled water. The PCR products were separated by electrophoresis through a 1% agarose gel, and purified using a Gel Extraction Kit (Takara) according to the manufacturer’s protocol. The purified products were then cloned into the pMD18-T Easy vector (Takara) and sequenced (Shangon, Shanghai, China).

### Sequence analysis

The open reading frames (ORFs) and encoded amino acid sequences of all genes were deduced using BioXM 2.6. Physicochemical properties of the deduced proteins were predicted using Protparam (http://www.expasy.ch/tools/protparam.html). Active sites of the protein sequences were analyzed by comparisons against the PROSITE database. The putative subcellular localizations of the candidate proteins were estimated by TargetP (http://www.cbs.dtu.dk/services/TargetP/) and Predotar (http://urgi.versailles.inra.fr/predotar/predotar.html).

### Phylogenetic analysis

Homologs of each member of the *Arabidopsis* FAD family were identified by BLASTP searches with datasets from Phytozome v10.3 (www.phytozome.net) and Peanut Genome Project (http://peanutbase.org/home) [34]. Only those sequences with an e-value less than e−^50^ were considered as members of the FAD family. In each tree, gene sequences were displayed using the nomenclature with the following abbreviations: At, *Arabidopsis thaliana TAIR10*; Glyma, *Glycine max Wm82*.*a2*.*v1*; Ah, *Arachis hypogaea* L.; Ai, *Arachis ipaensis*; Ad, *Arachis duranensis*; LOC_Os, *Oryza sativa v7*.*0*; Bradi, *Brachypodium distachyon v2*.*1*; Si, *Setaria italic v2*.*1*; GRMZM, *Zea may 6a*; Phpat, *Physcomitrella patens v3*.*0*; Sm, *Selaginella moellendorffii v1*.*0*; Cre, *Chlamydomonas reinhardtii v5*.*5*; Vocar, *Volvox carteri v2*.*0*; Ol, *Ostreococcus lucimarinus V2*.*0*; Mp, *Micromonas pusilla RCC1545 v3*.*0*; Cs, *Coccomyxa subellipsoidea C-169 v2*.*0*. The other amino acid sequences beyond the 16 species were retrieved from NCBI (http://www.ncbi.nlm.nih.gov/). S3 Table provides a detailed description of the proteins used and the corresponding accession numbers. S4 Table provides the pairwise comparison of the FADs from cultivated and wild peanut varieties. Amino acid sequences were aligned using the ClustalX program with the implanted BioEdit [76]. The neighbor-joining (NJ) method in MEGA4 [77] was used to construct the phylogenetic tree. Bootstrapping with 1,000 replicates was used to establish the confidence limits of the tree branches. Default program parameters were used.

### Quantitative real-time RT-PCR

qRT-PCR analysis was performed using a LightCycler 2.0 instrument system (Roche, Germany). The actin 11 gene (*AhACT11*) was selected as the reference gene [44]. Seventeen pairs of gene-specific primers (S5 Table) were designed after analyses of the target genes’ sequences. qRT-PCR reactions were performed using the SYBR Premix Ex Taq polymerase (Takara) according to the manufacturer’s instructions. Each 20-μl reaction was comprised of 2 μl of template, 10 μl of 2× SYBR Premix, and 0.4 μl (200 nM) of each primer. The reactions were subjected to an initial denaturation step of 95°C/10 s, followed by 40 cycles of 95°C/5 s, 60°C/30 s and 72°C/10 s. A melting curve analysis was performed at the end of the PCR run over the 60–95°C range, increasing the temperature stepwise by 0.5°C every 10 s. The baseline and quantification cycle (CP) were automatically determined using the LightCycler Software. Zero template controls were included for each primer pair, and each PCR reaction was carried out in triplicate. The relative quantification method (delta-delta Cp) was used to evaluate quantitative variation [78].

### Heterologous expression of *AhFAD6* in *Synechococcus elongatus* (strain PCC 7942)

The *AhFAD6* in the pSyn_1 plasmid was transformed into the freshwater unicellular cyanobacterium *S*. *elongatus* (strain PCC 7942), using the natural transformation method according to the manual (Invitrogen, Carlsbad, CA, USA). Transformants were selected by screening for resistance to 10 μg/mL spectinomycin in BG-11 solid medium. Colony PCR was performed to screen the transformed *S*. *elongatus* colonies for full integration of the promoter and the gene of interest. The positive colonies were transferred into BG-11 liquid medium with 10 μg/mL spectinomycin and grown at 28°C for 30 days. Cells were harvested by centrifugation, washed three times with double-distilled water and used for the extraction of total fatty acids.

### Fatty acid extraction and analysis

Total fatty acids were extracted and transmethylated with methanolic HCl from algae cells according to Browse et al (1986) [79]. All samples were analyzed using a 7890A/5975C gas chromatography (Agilent Technologies, California, USA) equipped with a 5975C single quadrupole GC/MSD detector and an HP-INNOWAX capillary column (30 m × 250 μm × 0.25 μm). High purity nitrogen was used as the carrier gas with flow rate of 40 mL/min. The injector and detector temperatures were both 250°C, and the column temperatures were programmed from 150°C to 230°C. Measurements were performed using peak height area integrals expressed as a percentage of the total of all integrals. The experiment was carried out in triplicate.

### Heterologous expression of *AhSLDs* in *Saccharomyces cerevisiae*

The *AhSLDs* in the pYES2 plasmid were transformed into the auxotrophic *S*. *cerevisiae* strain INVSc1 (MATa his3-Δ1 leu2 trp1-289 ura3-52), using the polyethylene glycol/lithium acetate method according to the manual (Invitrogen, Carlsbad, CA, USA). Yeast cells transformed with an empty pYES2 plasmid were used as the negative control. The *AhSLDs*-transformed yeasts were grown at 30°C in SC-U containing 2% (w/v) glucose for 24 h, and expression was further induced by the addition of 2% (w/v) galactose and 1% (w/v) tergitol NP-40 (Sigma, Taufkirchen, Germany) for an additional 72 h at 20°C. Yeast cells were collected by centrifugation for 10 min at 2000 g and dried at 50°C.

### Long chain base (LCB) analyses

Pellets of wild-type and transformed yeast cells (100 mg of dried weight) were used to prepare the LCBs for subsequent RP-HPLC analysis as previously described [80]. Phytosphinganine (t18:0) (Sigma) was used as the internal standard for yeast sample analyses. Briefly, induced yeast cells (100 mg, dried weight) were grounded into a fine powder, and subjected to strong alkaline hydrolysis in 2 ml of 10% (w/v) aqueous Ba(OH)_2_ and 2 ml of dioxane for 16 h at 110°C. After hydrolysis, 2 ml of 2% (w/v) ammonium sulphate was added, and the liberated sphingolipid long chain bases were extracted with 2 ml of diethylether. The upper phase was removed to a second tube, dried under nitrogen, and derivatized with o-phthaldialdehyde (OPA) (Invitrogen, Carlsbad, CA, USA) as previously described [81]. Individual LCBs were separated by RP-HPLC (Waters alliance 2695–2475 multi λ fluorescence detector, Waters, Milford, USA) with Penomenex C18 columns (250 mm 4.6 mm, 5 mm). Elution was performed at 1.2 mL/min with 20% solvent RA (5 mmol/L potassium phosphate, pH 7.0), 80% solvent RB (100% methanol) for 9 min, increasing to 90% solvent RB by 32 min, returning to 80% solvent RB and re-equilibrating for 2 min. Fluorescence was excited at 340 nm and detected at 455 nm.

## Supporting information

S1 Fig Alignment of deduced amino acid sequences of stearoyl-ACP desaturase genes of peanut and *Arabidopsis*. Identical amino acid residues were highlighted in black. The conserved histidine motifs were highlighted in black boxes.
(TIF)

S2 Fig Alignment of the deduced amino acid sequences of membrane desaturase genes. Identical amino acid residues were highlighted in black. The conserved histidine motifs were highlighted in black boxes.
(TIF)

S3 Fig Alignment of the deduced amino acid sequences of trans Δ3 desaturase genes. Identical amino acid residues were highlighted in black. The conserved histidine motifs were highlighted in black boxes.
(TIF)

S4 Fig Expression analysis of several fatty acid desaturase genes of peanut using qRT-PCR in peanut leaves under salt, drought or ABA treatment. (EPS)

S1 Table Difference of closely related multiple *AhFADs*. (XLS)

S2 Table DNA sequences of oligonucleotide primers used for gene cloning and vector construction in this study. (DOCX)

S3 Table The fatty acid desaturase enzyme homologs used for the phylogenetic analyses. The table shows the species, gene names and accession numbers of the sequences used in the analyses.
(XLS)

S4 Table Pairwise comparison of the FADs from cultivated and wild peanut varieties. (XLS)

S5 Table DNA sequences of oligonucleotide primers used for qRT-PCR in this study. (DOCX)

## Abbreviations

ADS: Δ9 desaturase
DES: sphingolipid Δ4 desaturase
DesA: cyanobacterial Δ12 desaturase
DesB: cyanobacterial ω3 desaturase
DesC: cyanobacterial Δ9 desaturase
FAB2: stearoyl-ACP desaturase
FAD2: microsomal Δ12 desaturase
FAD3: microsomal ω3 desaturase
FAD4: trans Δ3 desaturase
FAD5: Δ7 desaturase
FAD6: plastidial Δ12 desaturase
FAD7: plastidial ω3 desaturase
FAD8: plastidial ω3 desaturase
MGDG: monogalactosyldiacylglycerol
PG: phosphatidylglycerol
SLD: sphingolipid Δ8 desaturase
