# Supplementary figures and images for: Isolation and functional analysis of fatty acid desaturase genes from peanut (Arachis hypogaea L.)
Source: PLoS One. 2017 Dec 15;12(12):e0189759. doi: 10.1371/journal.pone.0189759 (PMC5731756; doi:10.1371/journal.pone.0189759)

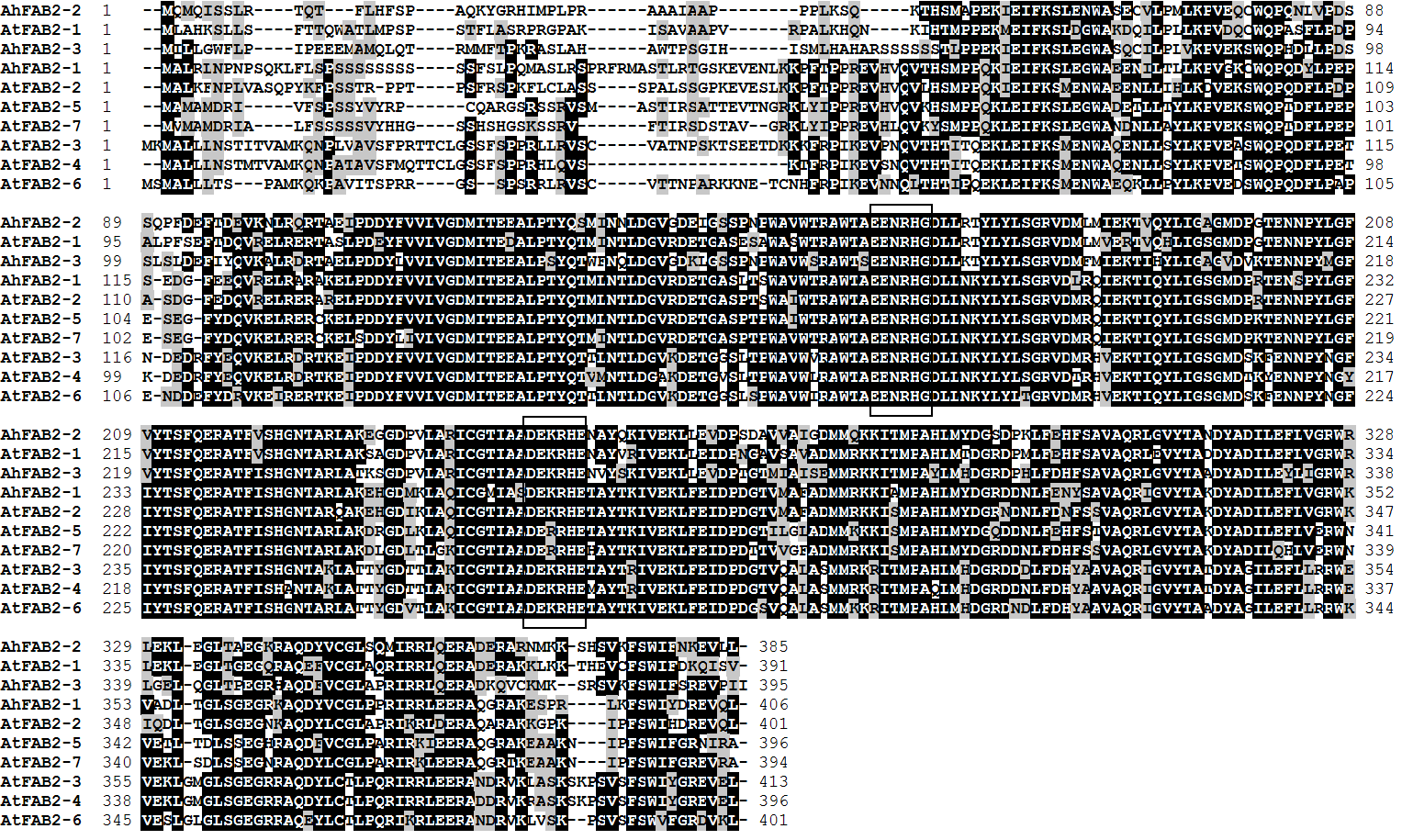

Supplement: S1 Fig — Identical amino acid residues were highlighted in black. The conserved histidine motifs were highlighted in black boxes. (TIF) [file pone.0189759.s001.tif]

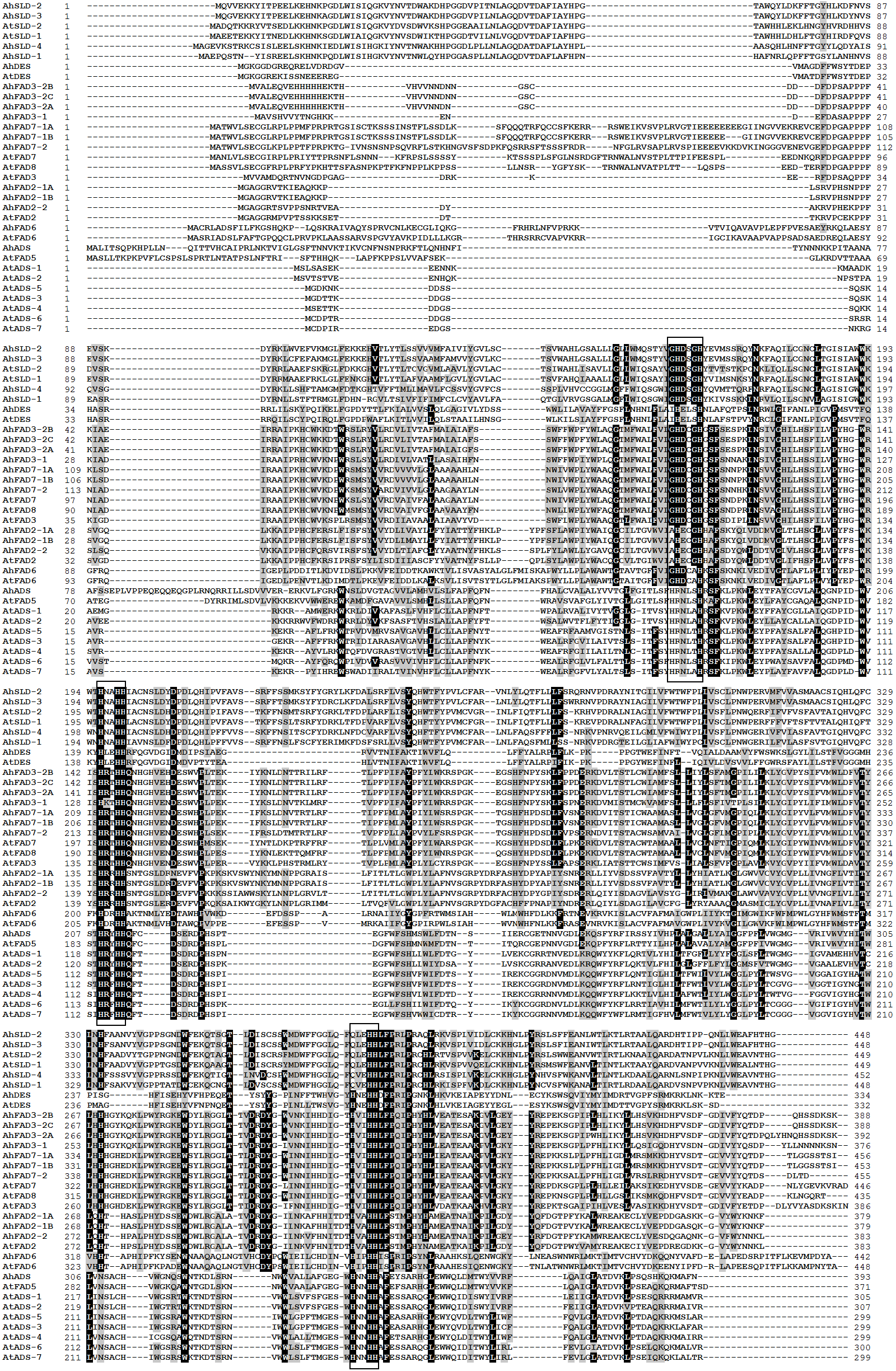

Supplement: S2 Fig — Identical amino acid residues were highlighted in black. The conserved histidine motifs were highlighted in black boxes. (TIF) [file pone.0189759.s002.tif]

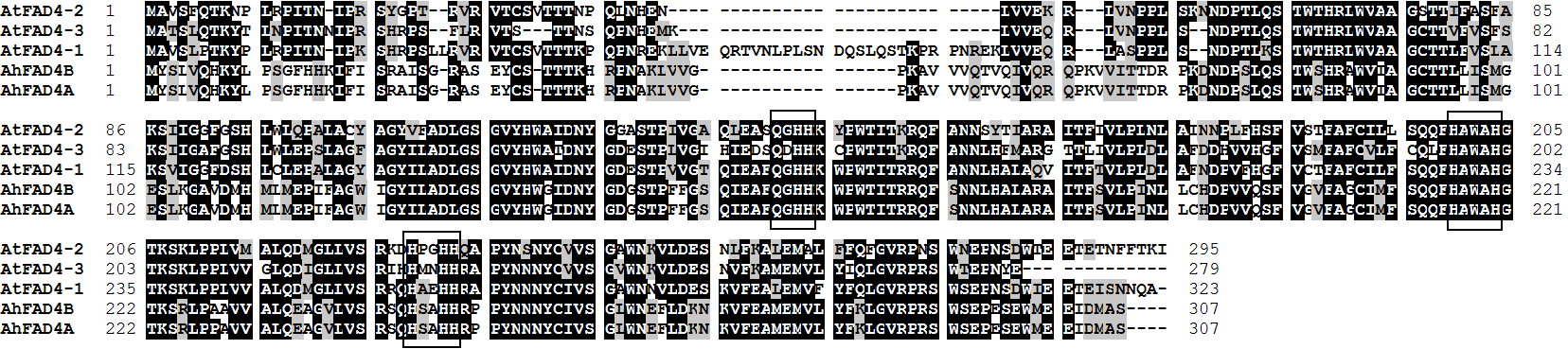

Supplement: S3 Fig — Identical amino acid residues were highlighted in black. The conserved histidine motifs were highlighted in black boxes. (TIF) [file pone.0189759.s003.tif]
