# Supplementary material for: Isolation and functional analysis of fatty acid desaturase genes from peanut (Arachis hypogaea L.)
Source: PLoS One. 2017 Dec 15;12(12):e0189759. doi: 10.1371/journal.pone.0189759 (PMC5731756; doi:10.1371/journal.pone.0189759)
Supplement: S2 Table — (DOCX) [file pone.0189759.s006.docx]

**S2 Table. DNA sequences of oligonucleotide primers used for gene cloning and vector construction in this study**

| Name | Oligonucleotide sequence 5’–3’ | Name | Oligonucleotide sequence 5’–3’ |
| --- | --- | --- | --- |
| Full-length cDNA sequence cloning | | | |
| \| FAB2-2-F \| \| --- \| | ATGCAAATGCAAATAAGTTC | \| FAB2-2-R \| \| --- \| | TCACAAAAGCACTTCCTTGT |
| FAB2-3-F | ATGATTCTCTTGGGTTGGTT | FAB2-3-R | TTATATTATGGGAACCTCTCTAC |
| FAD3-1-F | ATGGCAGTGTCCCATGTTGT | FAD3-1-R | CTAGTTGGACTTGTTATTGT |
| FAD3-2-F | ATGGTTGCCTTGGAACAAGT | FAD3-2-R | TCATTTAGACTTGTCCGAAGA |
| \| FAD4-F \| \| --- \| | ATGTACTCCTTAGTCCAACACAA | \| FAD4-R \| \| --- \| | TTAAGAAGCCATGTCAATCTCC |
| \| ADS-F \| \| --- \| | ATGGCTCTGATCACATCACA | \| ADS-R \| \| --- \| | TCAATTGAATGCCATTTTTT |
| \| FAD7-1-F \| \| --- \| | ATGGCAACATGGGTCTTATC | \| FAD7-1-R \| \| --- \| | TTAAATTGATGTAGAAGAGCCA |
| \| FAD7-2-F \| \| --- \| | ATGGCAACTTGGGTTTTATC | \| FAD7-2-R \| \| --- \| | TCAATCAGTGAGATCAGAGT |
| SLD-2-F | ATGCAGGTTGTTGAGAAGAA | SLD-2-R | TCAACCATGAGTGTGGAAAGC |
| SLD-3-F | ATGCAGGGGGTTGAGAAGAA | SLD-3-R | TCAACCATGAGTGTGGAAAGC |
| SLD-4-F | ATGGCTGGTGAGGTAAAATC | SLD-4-R | TCATCCATGAGTGTTAACGG |
| \| DES-F \| \| --- \| | ATGGGGAAAGGAGGTGACG | \| DES-R \| \| --- \| | CTATTCAGTTTTCTTGTTTAGCTT |
| Vector construction | | | |
| \| pYFAD6-F \| \| --- \| | \| ATAAAGCTTATGGCTTGCAGGCTTG \| \| --- \| | \| pYFAD6-R \| \| --- \| | \| TAAGGATCCTCAGGCATAATCAGGC \| \| --- \| |
| SLD-1yeast-F | TAGGATCCATGGCGGAACCACAATC | SLD-1yeast-R | CACCTCGAGTCATCCATGAGTGTTAAC |
| SLD-2yeast-F | TAGGATCCATGCAGGTTGTTGAGAAG | SLD-2yeast-R | TTGAATTCTCAACCATGAGTGTGGAA |
| SLD-3yeast-F | TTAAAGCTTATGCAGGGGGTTGAGAAG | SLD-3yeast-R | TTGAATTCTCAACCATGAGTGTGGAA |
| SLD-4yeast-F | TAGGATCCATGGCTGGTGAGGTAAAA | SLD-4yeast-R | CACCTCGAGTCATCCATGAGTGTTAAC |
