# Supplementary material for: Isolation and functional analysis of fatty acid desaturase genes from peanut (Arachis hypogaea L.)
Source: PLoS One. 2017 Dec 15;12(12):e0189759. doi: 10.1371/journal.pone.0189759 (PMC5731756; doi:10.1371/journal.pone.0189759)
Supplement: S5 Table — (DOCX) [file pone.0189759.s009.docx]

**S5 Table. DNA sequences of oligonucleotide primers used for qRT-PCR in this study**

| Name | Oligonucleotide sequence 5’–3’ | Name | Oligonucleotide sequence 5’–3’ | PCR efficiency  (%) | Regression coefficient (R^2^) |
| --- | --- | --- | --- | --- | --- |
| qACT11-F | TTGGAATGGGTCAGAAGGATGC | q ACT11-R | AGTGGTGCCTCAGTAAGAAGC | 95 | 0.996 |
| qFAB2-1-F | CGGTTAGGTCTGCCACCTTC | qFAB2-1-R | ACGCCACGAGACTGCATACA | 95 | 0.993 |
| qFAB2-2-F | TCTTGGAGTTCCTTGTTG | qFAB2-2-R | ACGCTATGTGACTTCTTC | 90 | 0.996 |
| qFAB2-3-F | TCATCATCATCATCAACATTAC | qFAB2-3-R | CTATCTCTTAGAGCCTTCAC | 90 | 0.999 |
| qFAD2-1-F | TGGTTAAGAGGAGCATTG | qFAD2-1-R | ATGAGGCATTGTTGAGAA | 95 | 0.996 |
| qFAD2-2-F | CCTCACACTCACTATTACCCTCAC | qFAD2-2-R | TGACAAGACGGATAAGACCATAGG | 94 | 0.983 |
| qFAD3-1-F | AAGGAATGGAGTTATCTAAGG | qFAD3-1-R | GCAAGTGATAGTGAGGAAT | 94 | 0.993 |
| qFAD3-2-F | AGTAGAACATCATCATCATCA | qFAD3-2-R | TCATCATCACAAGAACCAT | 91 | 0.998 |
| qFAD4-F | GACAACTATGGTGATGGT | qFAD4-R | ATAGGAAGCACTGAGAATG | 94 | 0.995 |
| qADS-F | CTGTGATTGGTCTTGGAT | qADS-R | CTGTTATTGGCTTCTTGTTAT | 90 | 0.997 |
| qFAD6-F | TCCATATTCCGCACCACATATCC | qFAD6-R | TTGTCTTCATCAGTCTCCAGTTCC | 91 | 0.996 |
| qFAD7-1-F | GAGGAAGAAGAAGAAGGAAT | qFAD7-1-R | CCAAGAACAACAACAACAT | 94 | 0.998 |
| qFAD7-2-F | TGAAGAAGAGGAAGTGAAG | qFAD7-2-R | TCCAAGAACAACAATTACATC | 91 | 0.999 |
| qSLD-1-F | TCTTGATGTGAGTTGTTCTTCTTGG | qSLD-1-R | GCGTCCGAATCGTGAGAGC | 91 | 0.997 |
| qSLD-2-F | GTTCATCGCTTATCATCCT | qSLD-2-R | GAGACCTCAGAGACATTG | 95 | 0.995 |
| qSLD-3-F | TCAGCCAATGTGTATGTT | qSLD-3-R | CAAGAGATGTCCAGTGTC | 95 | 0.998 |
| qSLD-4-F | AACACAACAAGATAGAGGAT | qSLD-4-R | AGGTTAAGCAATGGAAGG | 98 | 0.998 |
| qDES-F | ACTTCACTGTTCAGATAGC | qDES-R | AGAGGTCCATAGTAAGAGTAT | 95 | 0.992 |
